# Excision of the Elbow-Joint in a Case of Caries of the Articular Extremities of the Bones

**Published:** 1846-09

**Authors:** Gurdon Buck

**Affiliations:** Surgeon to the New York Hospital


### RECORD OF MEDICAL SCIENCE.

*Excision of the Elbow-Joint in a case of Caries of the Articular Extremities of the Bones*, by GURDON BUCK, JR., M. D., Surgeon to the New York Hospital.—Barney Foley, born in Ireland, aged 25 years, a steamboat fireman of temperate habits and good constitution, was admitted June 6, 1844, into Ward No. 3, Marine Building, New York Hospital, with inflammation of the right elbow-joint, of nearly two years' duration, that originated without injury or other known cause, while engaged in his occupation as fireman.

The only previous ailment he remembered to have had, was stiffness with contraction of the right knee-joint, at the age of fourteen, which was relieved by blisters.

The present affection commenced with a swelling between the olecranon and the the outer condyle, attended with stiffness and slight pain.

On being punctured, a glairy fluid with lumps of solid substance was discharged.

Patient has, with occasional interruptions, been able to use the limb, though the joint has continued stiff, and its motions have been impaired. Medical treatment has been employed for a year past, such as blisters and cupping, of which numerous marks are visible about the joint. Till recently patient has suffered but little pain, and his general health has continued good.

On commencing my attendance, the 1st July, the condition of the limb was as follows:—A uniform swelling involves the elbow, tapering off to the middle of the fore-arm below, and of the arm above, where the limb becomes small and wasted. The prominences of the olecranon and condyles, as well as the anterior fold of the joint, are obliterated. The swelling is formed by thickening and induration of the soft parts, the integument covering which, particularly over the posterior part, has lost much of its suppleness and mobility. Pressure over the olecranon and condyles causes pain. Synovial fluid is discharged from two openings, one of which is situated at the outer edge of the ulna, three fingers' breadth below the olecranon, the other at an inch above the inner condyle; the former leads to rough bone at the head of the radius, the latter, though it extends more than an inch inwards, does not communicate with exposed bone.

The habitual position of the limb is that of incomplete extension. A slight degree of flexion from this position is admissible, as well as partial pronation and supination, all of which are attended with pain. No sensation of roughness is perceptible in performing these motions.

The surface of the swelling is pale, the temperature above that of the rest of the limb. Two issues have been established near the outer condyle since his admission, and an attempt made to preserve the limb flexed at a right angle, it being the most favorable position in which to allow anchylosis to take place. This, however, had to be abandoned in consequence of the increased pain and inflammation that followed.

Patient's general health is good, pulse sixty-two, tongue somewhat furred, bowels good.

July 25.—Preparatory to the operation to be performed this day, the issues have been allowed to heal up. The swelling about the joint has rather diminished.

The upper opening extends two inches downwards and outwards, and now communicates with denuded bone.

At a distance of two fingers' breadth above the condyles, the os brachii appears to be uneven and enlarged. The patient's general condition continues favorable ; appetite good, and bowels regular.

*Operation.*—The tourniquet having been applied at the insertion of the deltoid muscle, and the patient placed in the recumbent posture, on his left side, with the head and shoulders elevated, and his back towards the operator, a longitudinal incision, six inches in length, was made over the olecranon, extending to a distance above and below it, and penetrating to the bone. The triceps muscle and tendon thus split, were raised toward the outer condyle, care being taken to keep close to the bone and avoid dividing the connections of the tendon with the aponeurosis of the fore-arm. The same course was pursued in the dissection toward the inner condyle, the ulnar nerve being drawn inwards to prevent its being wounded. The integuments and subjacent aponeurosis were next raised on either side, below the olecranon, the olecranon itself denuded, and more than an inch of this process removed with the amputating saw. The edges of the wound being drawn forcibly to either side, the articular extremity of the os brachii was detached from its connections by dividing the lateral ligaments and the muscles arising from the condyles, which allowed it to be projected from the surrounding soft parts, while a transverse section was made above the condyles, separating a portion of an inch and a half in length. The head of the radius was now ascertained to be rough and denuded, as well as the smaller sigmoid cavity in which it rotates upon the side of the ulna. The division of the annular ligament allowed the head to be cleared from the soft parts sufficient to excise it at its neck. An additional portion of the ulna, including the coronoid process and lesser sigmoid cavity, remained still to be excised, which was effected by carefully dissecting up the insertions of the brachialis anticus muscle as far as was necessary, and then making a section from before, backwards, with a metacarpal saw applied just below the coronoid process. The rough angular edges of bone were then pared away. Several ligatures were applied to small vessels, and the edges of the wound brought together with seven sutures, between which adhesive straps were applied, passing half round the limb.

The diseased part presented the following appearances. A greyish, jelly-like substance covered the synovial surfaces, being most abundant where the synovial membrane passes from the bone and lines the ligaments.

This morbid product could be easily scraped off, and brought to view the spongy tissue of the bone, destitute of cartilage as well as compact outer shell. The spongy tissue thus exposed, was red and softened, and could easily be penetrated by the scalpel. Small patches of cartilage still remained at the margins of the articular surfaces, which were only loosely adherent to the subjacent spongy tissue, the exterior shell having been absorbed.

Between the outer condyle and the small head of the humerus, a deep ulcerated excavation, capable of containing a white bean, had been formed. The surface of the posterior fossa of the humerus, lodging the olecranon, was rough and bare. The articular surface of the ulna presented similar appearances to the os humeri, with two or three superficial excavations at the bottom of the greater sigmoid fossa.

The surface of the coronoid process and of the olecranon, as well as the inner margin of the sigmoid fossa, are studded with spiculæ of newly formed bony matter.

The head of the radius was more completely deprived of cartilage and bony shell than the parts already noticed, and their place supplied by a thick layer of gelatinous deposit.

After the dressing of the wound, the limb was placed nearly at a right angle, on a flat splint padded with cotton, with a joint at the elbow allowing it to move edgewise. By means of this splint the arm was suspended from the cealing after the patient was conveyed to his bed. This arrangment afforded great comfort; the limb swinging clear of his body, allowed him to vary his position within certain limits, an advantage he could not enjoy in any other way, compatible with perfect rest of the new joint. Sixty drops tinct. opii were then given.

At evening, about five hours after the operation, the patient began to suffer severe pain in the elbow, and the wound appeared distended, with blood, which oozed from between the sutures. Thirty drops tinc. of opium, spirits mindereri ℥ss., were ordered to be repeated every two hours.

July 26. Patient suffers less pain, swelling of the elbow has increased, with tension and slight lividity of the surface, oozing of blood continues, removed two sutures and an intermediate strap from the middle of the wound, and readjusted the bandages that had become too tight; pulse ninety-seven. Ordered light poultice of flaxseed meal.

At 6 P. M. Oozing of blood has ceased, complains of pain in the left hypochondrium, extending to the right side, no evacuation for forty-eight hours.

℞ Tinct. Opii gtts. xxx.

Ol. Ricini ℥i.

At 10, P. M. to take his anodyne draught of Tinct. Opii gtts. xxx. Aq. Menth. pip. ℥ss.

July 27. Has passed a good night, general appearance is improved, free from pain in the abdomen, pain in the arm slight, serous fluid oozes from the wound, swelling is not increased, pulse ninety-four, soft, tongue coated with whitish fur, bowels still confined.

℞. Infus. Sennæ comp.

July 28. Passed a good night after taking the same anodyne as the night previous, has had two evacuations following a laxative enema, pulse seventy-four ; removing the dressings, the elbow appeared well, tension has diminished, swelling the same, reapplied only two adhesive straps, continued poultice.

29. Doing well, suppuration is commencing, five ligatures came away, omitted adhesive straps, continued poultice, bowels confined, pulse seventy-two ; ordered Mistura Eccoprotica.

30. Pulse sixty-eight, one evacuation from the bowels, tension of the elbow still further diminished as well as redness of the surface, one ligature came away, and removed one suture from each extremity of the wound.

31. Doing well, bowels confined, pulse seventy-four, removed two ligatures, and the remaining sutures were removed ; ordered pil. cathart. three.

Aug. 1. Tension and redness of the elbow have disappeared ; the three remaining ligatures came away.

3. Passed a good night, pulse 64, tongue is cleaning off and appetite returning ; union by the first intention is fully established, except the middle of the wound, which had been kept open to facilitate the discharges ; the suppuration is moderate and of healthy appearance, redness has entirely disappeared and swelling further diminished ; omitted poultice and dressed with simple cerate, and a bandage loosely applied ; allowed more generous diet.

Aug. 12. Continues doing well, suppuration has at no time exceeded one ounce in twenty-four hours, and has now almost disappeared. A slight synovial discharge escapes from the old opening, below the head of the radius. For two days past, patient has been allowed to walk about, with the arm supported on a splint in a sling. Gentle passive motion has been employed for about a week past.

21. Wound has nearly healed. Synovial discharge continues as already noticed ; suffers no pain in the elbow from motion, nor is any sensation of rough bone perceptible. The new joint has acquired considerable firmness, and the extent of motion of which it is susceptible, is from almost complete extension, to an acute angle, allowing the hand to be brought to the mouth, and also a good degree of pronation and supination ; the latter motion patient performs without any assistance.

Sept. 17. Patient has gradually acquired the use of his arm, to the extent of being able to flex the elbow, and bring his hand to his head. A slight synovial discharge continues from two remaining openings ; for some time past has suffered from pain in the elbow, referred to the ends of the bones. No appearance of inflamation, or feeling of roughness, exists.

Complained for two days past of headache, sense of weight in the stomach, sickness and occasional chillness, with loss of appetite. Twenty-four hours after the onset of these symtoms, erysipelas showed itself at the elbow, extending half way to the shoulder above, and to the middle of the fore-arm below, tongue covered with a thick yellowish brown fur, pulse moderately accelerated ; ordered

| | | |
|---|---|---|
| Tart. Emetic., | gr. | ij. |
| Calomel, | gr. | x. |
| Ipecacuanba, | ℈ | j. |

to be followed by comp. infus. sennæ ; warm infusion of opium to be applied to the limb.

Sept. 19. Erysipelas is fading, patient free from fever, bowels confined ; ordered Seidlitz powders every two hours till they operate.

The further progress of this case continued favorable, with unimportant exceptions. One or two small collections of matter formed near the elbow, but after being opened, discharged and soon healed.

The limb gradually acquired increased strength and facility of motion. The power of grasping bodies with the hand, which had been very much impaired by the disease, was recovered in a good degree ; pronation and supination could be performed almost to the original extent ; patient was able, unassisted, to raise the hand to the head, and could handle a broom in sweeping, carry half a pail of water, and perform other useful functions to his own great satisfaction. He was also sensible of progressing improvement in his limb. His general health was good ; the only symptom of which he complained, was a stricture across the chest. He was discharged this day, Feb. 1, 1845, to return to his family at Buffalo.

*Remarks.*—The reason for preferring, in this case, the operation with a single longitudinal incision, to an incision in the form of the letter H or to the crucial incision, more generally recommended by authorities, was with a view to preserve undivided the lateral connections of the tendon of the triceps with the fascia investing the fore-arm, which must necessarily be sacrified by either of the other modes of operating. The important advantage of so doing was, that a point of action would be preserved for the triceps muscle, which might compensate in a good degree for the sacrifice of its connection with the olecranon. All the other essential objects of the operation, such as the preservation of the ulnar nerve, &c., were attained by this method, without any increased disturbance of the soft parts, or embarrassment in its several stages. The subsequent progress of the case could scarcely have been more favorable under any circumstances, and from the experience derived from this case, together with one reported in No. VIII., of New York Medical and Surgical Journal of April, 1841, in which the H incision was employed, I should be disposed decidedly to give preference to the method adopted in the present instance. Rather more time, perhaps, is required for this operation, but the great advantage already noticed should certainly be considered more than an equivalent.

The subsequent condition of the patient after the lapse of more than a year from the operation, was ascertained by my brother, Mr. Edward Buck, who at my request saw him, while on a visit at Buffalo, and communicated to me the particulars of his case in a letter of which the following is an extract:

*Buffalo, Nov.* 21, 1845.

"I found your man, Barney Foley. The doctors (I inquired of three) had a general impression like yourself, that he kept a grocery near the water; for specific directions, I was indebted to an Irishman, who came shoeless out of a grog-shop. Barney and his wife were very cordial.

"I made him go through the evolutions with his arm. I do not see but he can perform all the functions, viz: scratch his head, put his hand in his vest pockets, (breeches of course,) handle a knife and fork, carry a pail of water, write, but he is not gifted in raising weights, or in tying his cravat; but as he wears no collar except on Sundays, this is a small inconvenience. He has no pain in the arm or soreness of any kind, but when a storm comes on, the arm is weaker than usual.

"Shortly after he returned to Buffalo, there was a swelling, as you predicted there would be; he went to a doctor here, who told him that you had left a piece of the bone in the arm, and he would have to suffer an amputation after all. Barney thought this an insult to you, and went to another doctor, who punctured it. That answered the purpose, and his arm is now healed up and sound, though scarred. He strained it some time last summer, falling into the water and getting pulled out. Soon after the operation he had a stricture across his chest, but that has left him too."—*New York Jaurnal of Medicine.*

